# Comparison of Ropivacaine Plus Dexmedetomidine and Ropivacaine Plus Magnesium Sulfate Infiltration for Postoperative Analgesia in Patients Undergoing Lumbar Spine Surgeries

**DOI:** 10.7759/cureus.36295

**Published:** 2023-03-17

**Authors:** Manoj Kumar, Rakesh Bahadur Singh, Jai Prakash Vikal, Jay Brijesh Singh Yadav, Dheer Singh

**Affiliations:** 1 Anesthesiology, Uttar Pradesh University of Medical Sciences, Etawah, IND

**Keywords:** spine surgeries, postoperative pain, infiltration, magnesium sulphate, dexmedetomidine, ropivacaine

## Abstract

Background: Acute pain after lumbar spine surgery is due to soft tissue and muscle separation at the operation site. Local anesthetic wound infiltration is a safe and effective method for postoperative analgesia following lumbar spine surgery. In this study, we aimed to investigate and compare the efficacy of ropivacaine plus dexmedetomidine and ropivacaine plus magnesium sulfate for postoperative analgesia in lumbar spine surgeries.

Materials and method: This prospective randomized study was conducted on 60 patients, aged between 18 and 65 years, either sex, American Society of Anesthesiologists classification I and II patients scheduled for single-level lumbar laminectomy. Patients were randomly allocated into two groups 30 patients each. Twenty to 30 minutes before skin closure and after hemostasis was achieved, the surgeon infiltrated 10 mL of study drugs into paravertebral muscles on each side. Group A received 20 mL of 0.75% ropivacaine plus dexmedetomidine and group B received 20 mL of 0.75% ropivacaine plus magnesium sulfate. Postoperative pain was assessed by the visual analog scale at 0 minute (immediately after extubation), 30 minutes, 1st hour, 2nd hour, and thereafter at 4th hour, 6th hour, 12th hour, and 24th hour. Time to rescue analgesia, total analgesic consumption, hemodynamic variables, and complications if any were recorded. Statistical analysis was done using SPSS version 20.0 (Armonk, NY: IBM Corp.).

Results: The time to first requirement of analgesia in postoperative period was significantly longer in group A (10.05 ± 1.62 hours) than in group B (8.07 ± 1.83 hours) (p < 0.001). Total analgesic consumption was significantly higher in group B (197.50 ± 36.76 mL) compared to group A (142.50 ± 22.88 mL) (p < 0.001). Heart rate and mean arterial pressure were significantly lower in group A compared to group B (p < 0.05).

Conclusion: Local infiltration of surgical site with ropivacaine plus dexmedetomidine provided better pain control than ropivacaine plus magnesium sulphate infiltration and is safe and effective analgesia for patients undergoing lumbar spine surgeries in postoperative period.

## Introduction

Postoperative pain involves nociceptive, neuropathic, and inflammatory pain responses that are directly proportional to the number of vertebral levels involved in the lumbar spine surgeries [1]. Patients usually complain of severe pain during the initial 12 hours in postoperative period. This pain surges considerably with mobilization due to reflex contraction of the paraspinal muscles, triggered primarily by the pain at the wound site. During the subsequent 48-72 hours, back pain is generally moderate at rest, aggravated on movement, and produces discomfort during postoperative period. Delayed mobilization contributes to increased risk of medical complications, such as pneumonia, deep vein thrombosis, urinary tract infection, and psychological stress [2].

Multimodal pain control therapy is nowadays advocated [3]. Various adjuvants such as clonidine, magnesium, and dexmedetomidine added to local anesthetics for infiltration have shown encouraging outcomes [4]. Local anesthetics infiltration at the wound site is an attractive strategy since it is effective and side effects are negligible [5]. Nowadays, there is a tendency towards favoring ropivacaine over other local anesthetic agents due to its longer duration of action and improved safety profile [6]. The pain relief is present only till the effects of local anesthetic action persist. Efforts are being made to lengthen the duration of action of local anesthetic infiltration in the wound area. Dexmedetomidine, an alpha-2 adrenoceptor agonist is one such agent which can potentiate and extend the duration of local anesthetic for relief of pain. Numerous literatures are available regarding the role of dexmedetomidine as an adjuvant to local anesthetics, such as ropivacaine for peripheral nerve blocks and intrathecal administration as a part of regional anesthesia [7]. Magnesium sulfate is a physiological antagonist at N-methyl-D-aspartate (NMDA) receptor [8]. NMDA receptors are present in the central nervous system as well as in the peripheral tissues, such as skin and muscles. Dexmedetomidine, a potent alpha-2 adrenergic receptor agonist is approximately eight times more selective at receptor sites than clonidine [9]. Dexmedetomidine has also been used as an additive to local anesthetics agents for various types of nerve blocks [10].

However, there are no clinical studies available to compare the efficacy of magnesium sulfate combined with local anesthetic drugs, such as bupivacaine and ropivacaine, for wound site infiltration in lumbar spine surgeries. The objective of this study was to investigate and relate the effects of wound site infiltration while using ropivacaine plus dexmedetomidine and ropivacaine plus magnesium sulfate in patients with lumbar spine surgeries.

## Materials and methods

After approval from the Ethical Committee of our institution, Uttar Pradesh University of Medical Sciences (UPUMS), Etawah, India (ref. no. 1883/UPUMS/Dean(M)/Ethical/2020-21) and written and informed consent, this prospective randomized double-blind study was conducted in the Department of Anesthesia, UPUMS, Etawah, India.

Inclusion and exclusion criteria

Patients aged between 18 and 65 years, either sex, American Society of Anesthesiologists (ASA) physical status classification I and II scheduled for elective single-level lumbar laminectomy under general anesthesia were enrolled in the study. Patients who did not provide their consent, hepatic or renal failure, cardiopulmonary disease, mental retardation or neurological deficits, psychiatric disorders, history of allergy to ropivacaine, dexmedetomidine, and magnesium sulfate were excluded from the study.

Sample size calculation and randomization

Taking into consideration an alpha error of 0.05 and power of study as 95%, the projected sample size was revealed to be 30 patients per group. The following sample size equation was used: n = (Z _α/2_ + Z_β_)^2 ^× 2σ^2 ^/ (µ_1_ - µ_2_)^2^. Here, n = sample size per group. Z_α/2_ = standard normal z-value for significance level α =0.05, Z_β_ = standard normal z-value for the power of 80%, which is 0.80, µ_1 _= mean of affect at baseline, µ_2_ = mean of affect after drug administered, and σ = standard deviation.

A random number table for 60 patients was prepared and using sequentially numbered opaque sealed envelopes patients were randomly assigned into two groups. Group A (n = 30) was given 15 mL of 0.75% ropivacaine + 50 µg dexmedetomidine equivalent to 0.5 mL + 4.5 mL normal saline. Group B (n = 30) was given 15 mL of 0.75% ropivacaine + 500 mg magnesium sulfate equivalent to 1 mL + 4.0 mL normal saline. The total volume of the solution infiltrated was 20 mL in both groups.

All patients were explained the day before surgery on how to inform about the intensity of pain using a visual analog scale (VAS), a score of 0 to 10, where 0 = no pain, 5 = distressing pain, and 10 = unbearable pain. Patient satisfaction data was assessed using Likert verbal rating scale, where 1 = extremely dissatisfied, 2 = dissatisfied, 3 = somewhat dissatisfied, 4 = undecided, 5 = somewhat satisfied, 6 = satisfied, and 7 = extremely satisfied.

Procedure

All the patients received tablet alprazolam 0.25 mg and pantoprazole 20 mg orally the night before surgery. All patients were kept fasted for 6-8 hours before surgery. In the operation theatre, peripheral vascular access was obtained with an 18-gauge intravenous cannula and lactated ringer’s solution was commenced at 10 mL/kg. ASA standard monitors were attached which includes non-invasive blood pressure (NIBP), pulse rate (PR), electrocardiography (ECG), peripheral saturation of oxygen (SpO_2_), and capnography (EtCO_2_) and monitoring was conducted throughout the intraoperative and postoperative period. Participants were premedicated with injection glycopyrrolate 200 µg, injection midazolam 0.05 mg/kg, injection fentanyl 2 µg/kg, intravenously. After 3 minutes of preoxygenation, injection propofol 2 mg/kg or in a dose sufficient for loss of verbal commands was administered. Direct laryngoscopy and endotracheal intubation were aided with injection vecuronium 0.1 mg/kg and intubation was performed with cuffed endotracheal tube size 7.0-7.5 mm in females and 8.0-8.5 mm in male patients. Intraoperative anesthesia was maintained with 66% N_2_O, 33% O_2_, and isoflurane (0.6-1%) using controlled ventilation. Supplemental neuromuscular blockade was achieved with injection vecuronium in a divided dose of 0.02 mg/kg body weight. Intraoperative analgesia was administered using paracetamol 15 mg/kg intravenously. Intraoperative monitoring was done every 5 minutes. At the end of the surgical procedure and before the skin closure, the surgeon infiltrated 10 mL of study drugs into paravertebral muscles on each side.

At the end of surgical procedure, residual neuromuscular blockade was reversed with the injection neostigmine 0.05 mg/kg and glycopyrrolate 0.01 mg/kg. Both the study patients and the independent person who recorded the data were blinded to the group allocation. Postoperative pain and satisfactions score were evaluated using visual analog scale (VAS) and Likert verbal rating scale, respectively. VAS score observed first at 0 minute (immediately after extubation), 30 minutes, 1st hour, 2nd hour, and thereafter at 4th hour, 6th hour, 12th hour, and 24th hour. The time of first analgesic request was considered from the study drug infiltrated to the point of first analgesic demand in the postoperative period. When VAS score reached > 4, injection diclofenac 1.5 mg/kg body weight was administered as rescue analgesic over 15 minutes and repeated if needed. The primary outcome variable in this study was the pain score using VAS. The secondary outcome variables were the time to rescue analgesia, total analgesia consumption, and hemodynamic variables viz. heart rate, mean blood pressure, and respiratory rate in 24 hours. Patients were also observed for any complications in the postoperative period.

Statistical analysis

Data were represented as mean±SD (standard deviation) and percentages (%). Statistical analysis of quantitative variables was done and compared between groups using unpaired t-test and within groups using paired t-test. Statistical analysis of qualitative variables was done and compared using chi-square/Fisher's exact test. P-values < 0.05 were considered statistically significant. Statistical analysis was performed using SPSS version 20.0 (Armonk, NY: IBM Corp.).

## Results

**Figure 1:**
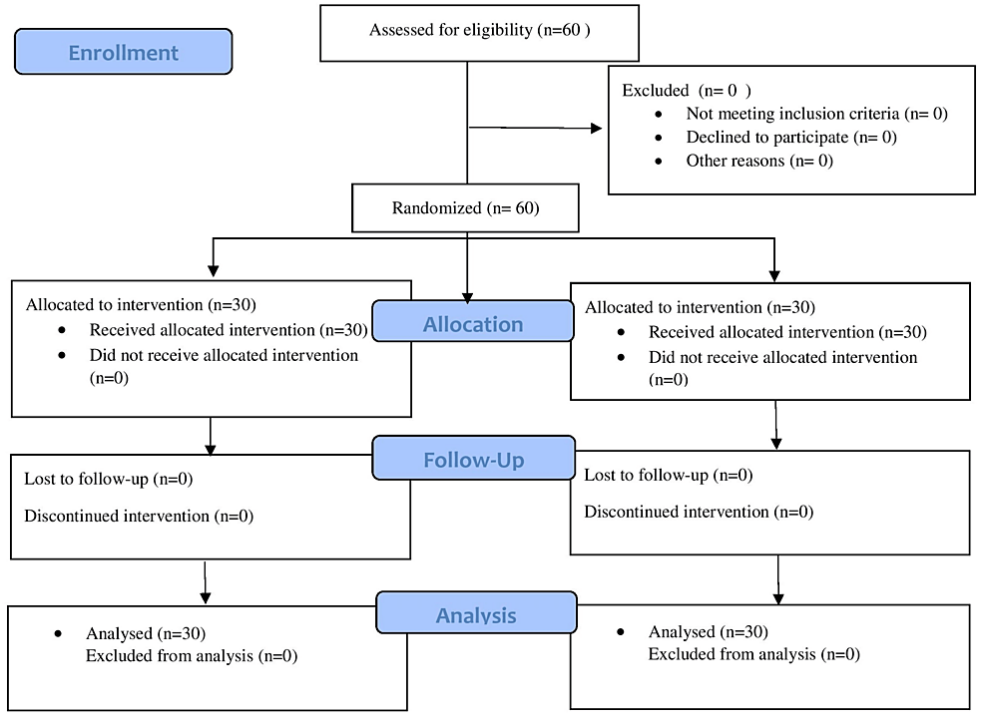
Consolidated Standards of Reporting Trials (CONSORT) flow diagram.

No patients were excluded from the study, 60 patients scheduled to undergo lumbar spine surgery were randomly divided into two groups of 30 patients each (Figure 1). The demographic variables (age, sex, weight, height) and ASA physical status classification were statistically not significant between study groups (p > 0.05) (Table 1).

**Table 1:** Demographic characteristics among the studied groups. ASA PS: American Society of Anesthesiologists physical status classification

Parameters	Group A (n=30)	Group B (n=30)	p-Value
Age (years)	38.17±14.63	35.17±13.68	0.208
Height (cm)	165.30±6.76	165.90±8.58	0.382
Weight (kg)	55.93±8.91	57.83±8.81	0.205
Sex (male/female)	25/5	22/8	0.174
ASA PS I/II	11/19	14/16	0.216
Duration of surgery (minutes)	121.50±5.28	121.67±3.56	0.443

**Figure 2:**
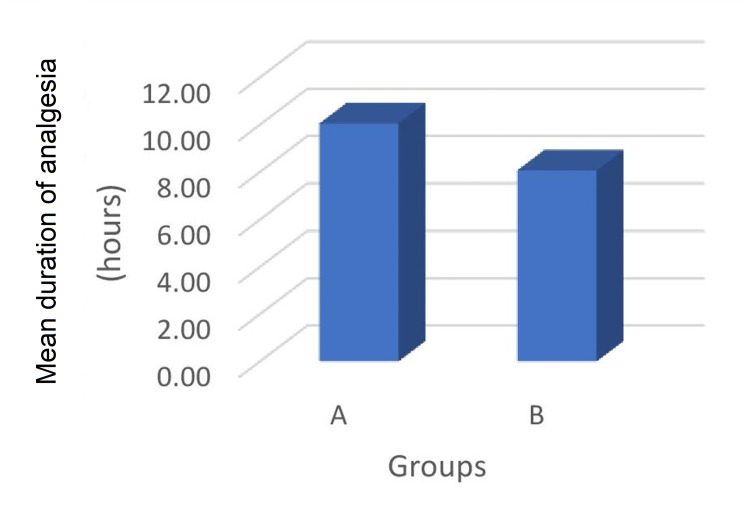
Comparison of mean duration of analgesia between the studied groups.

**Figure 3:**
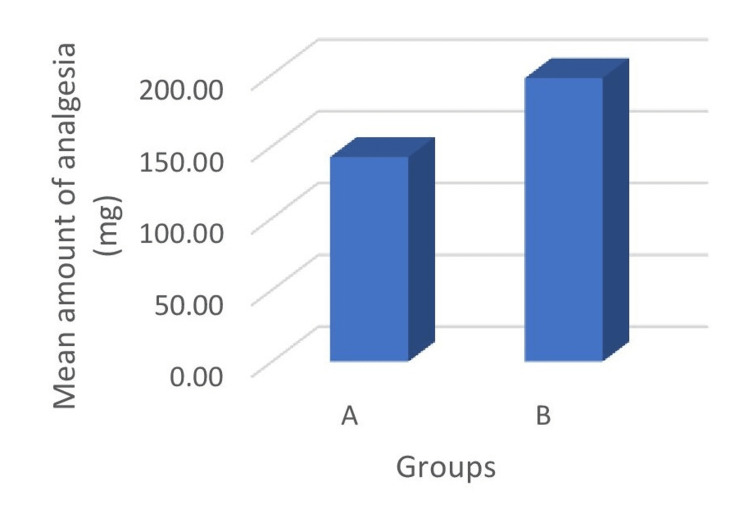
Comparison of total analgesic consumption between the studied groups.

The mean duration of time to rescue analgesia in group A (8.07 ± 1.83 hours) was longer in contrast to group B (10.05 ± 1.62 hours) and was observed to be statistically significant (p < 0.001) (Figure 2). The total analgesic consumption in 24 hours was 142.50 ± 22.88 mg and 197.50 ± 36.76 mg in group A and group B, respectively. On intergroup comparison, total analgesic consumption was less in group A than group B, and mean difference was observed to be statistically highly significant between the groups (p < 0.001) (Figure 3).

VAS score was comparable between the group at all time intervals except at 6th and 12th hour where at 6th-hour VAS score was higher in group B (4.67 ± 0.48) compared to group A (1.77 ± 0.43) (p < 0.001). VAS score at 12th hour was higher in group A (5.00 ± 0.00) compared to group B (2.53 ± 1.01), (p < 0.001) (Table 2).

**Table 2:** Comparison of visual analog scale (VAS) score among the studied groups.

VAS score	Group A (n=30) mean±SD	Group B (n=30) mean±SD	p-Value
Baseline (preoperative)	0.00±0.00	0.00±0.00	-
0 min (just after extubation)	0.00±0.00	0.00±0.00	-
30 minutes	1.57±0.50	1.43±0.50	0.155
1st hour	1.63±0.49	1.63±0.49	0.500
2nd hour	1.60±0.50	1.63±0.49	0.397
4th hour	1.83±0.46	1.70±0.47	0.135
6th hour	1.77±0.43	4.67±0.48	<0.001
12th hour	5.00±0.00	2.53±1.01	<0.001
24th hour	1.80±0.41	1.70±0.51	0.070

Table 3 shows that the mean satisfaction score by Likert verbal rating scale in group A and group B was 6.73 ± 0.45 and 6.23 ± 0.43, respectively. On intergroup comparison, patient's satisfaction score was higher in ropivacaine plus dexmedetomidine group compared to ropivacaine plus magnesium sulfate group and was observed to be highly significant (p < 0.001).

**Table 3:** Comparison of Likert verbal rating scale among the studied groups.

Likert verbal rating scale	Group A (n=30)		Group B (n=30)		p-Value
	n	%	n	%	
6	8	26.67%	23	76.67%	<0.001
7	22	73.33%	7	23.33%	
Total	30	100%	30	100%	-
Mean±SD	6.73±0.45		6.23±0.43		<0.001

**Figure 4:**
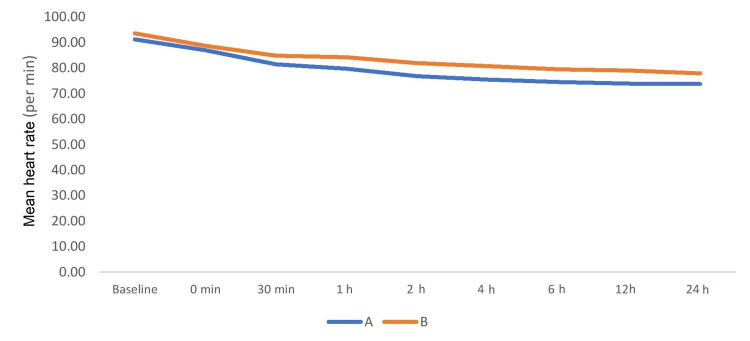
Comparison of mean heart rate between the study groups.

On intergroup comparison, mean heart rate values were comparable at the baseline between the groups (p > 0.05). After 30 minutes of extubation, mean heart rate was lower in group A (81.47 ± 6.34) compared to group B (84.87 ± 7.58) and was statistically significant (p < 0.05). Thereafter also heart rate remained lower in group A than group B and remained statistically significant between the groups at all time intervals during 24 hours (p < 0.05) (Figure 4).

**Figure 5:**
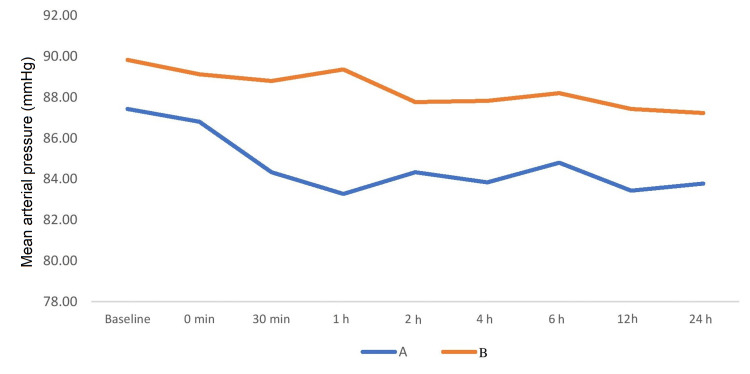
Comparison of mean arterial pressure among the groups.

**Figure 6:**
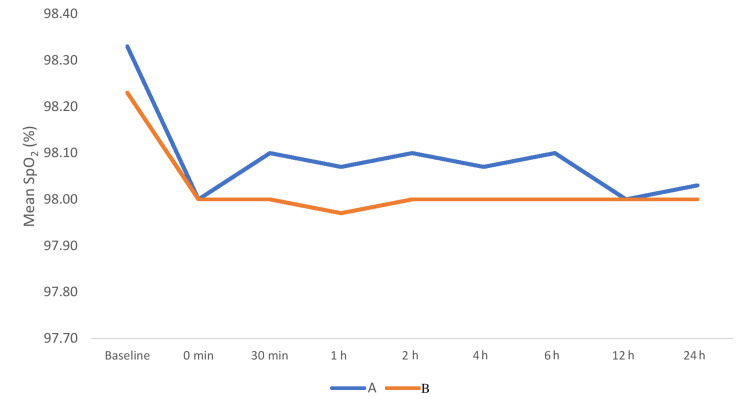
Comparison of peripheral saturation of oxygen SpO2 (%) among the groups.

During intergroup comparison, mean arterial pressure between the study groups was comparable at baseline (p > 0.05). After 30 minutes of extubation, mean arterial pressure was lower in group A (84.33 ± 4.39) compared to group B (88.80 ± 6.16) and was statistically significant (p < 0.05). Thereafter also mean arterial pressure remained lower in group A than group B and remained statistically significant between the groups at all time intervals during 24 hours (Figure 5). Figure 6 shows the comparison of peripheral saturation of oxygen between the groups. The groups were comparable in terms of SpO_2_ at all time periods (p > 0.05).

Adverse effects such as nausea, vomiting, allergic reaction, dry mouth, respiratory depression, and urinary retention were not reported in either group.

## Discussion

During lumbar spine surgeries, acute postoperative pain occurs due to stimulation of nociceptor and mechanoreceptors found in the vertebrae, intervertebral disc, nerve roots, dura, facet joint capsules, muscles ligaments, and fascial sheaths. These structures are innervated mainly by posterior rami of spinal nerve roots connected to the autonomic nervous system. Inflammation or mechanical compression of these structures leads to initiation of nociceptive impulse. The stimulation of these receptors led to sensitization of adjoining neurons. Substantial alternations occur over a period of time as nociceptive impulses from both the peripheral as well as central nervous systems get integrated causing central pain sensitization which can lead to exaggerated and prolonged postoperative pain or chronic pain syndromes.

This study was conducted to compare the efficacy of ropivacaine plus dexmedetomidine and ropivacaine plus magnesium sulfate infiltration for postoperative analgesia in patients undergoing lumbar spine surgeries. In our study, the demographic profiles (age, sex, weight, height), ASA physical status classification, duration of surgery, hemodynamic variables, visual analog scale for pain, Likert verbal rating scale for patient satisfaction score, duration of analgesia, and total analgesic consumption were recorded and compared. In our study, the demographic characteristics, duration of surgery, and SpO_2_ were comparable with respect to each other (p > 0.05). In our study, the mean VAS score was comparable between the groups at all time intervals except at 6th and 12th hour. At 6th hour, VAS score was higher in group B (4.67 ± 0.48) compared to group A (1.77 ± 0.43) (p < 0.001). At 12th hour, VAS score was higher in group A (5.00 ± 0.00) compared to group B (2.53 ± 1.01) (p < 0.001). Our observations were consistent with the study done by Deshwal et al. who compared the efficacy of dexmedetomidine plus ropivacaine versus ropivacaine alone in wound infiltration after lumbar discectomy [11]. The VAS score was significantly less in dexmedetomidine plus ropivacaine group when compared to ropivacaine alone infiltration group. Another study conducted by Kang observed the efficacy of dexmedetomidine added to pre-emptive ropivacaine infiltration for inguinal herniorrhaphy [12]. The VAS score was lower in dexmedetomidine with ropivacaine group postoperatively. These observations were also in concordance with our study. Daiki et al. conducted a study to compare the postoperative analgesia after wound infiltration with dexmedetomidine and ropivacaine (RD) group versus ropivacaine (R) group in lumbar discectomies [13]. The VAS score was less in group RD in comparison to group R (p < 0.0001). Similar observations were also reported in our study. Singh and Prasad conducted a randomized controlled trial to observe the analgesic effect in patients who received bupivacaine and bupivacaine plus dexmedetomidine in wound infiltration for abdominal hysterectomy [14]. The pain score was lower in bupivacaine plus dexmedetomidine group when compared to bupivacaine alone group and was statistically significant (p < 0.01).

In our study, the duration of analgesia in group A was longer (10.05 ± 1.62 hours) compared to group B (8.07 ± 1.83 hours) and observed to be statistically significant (p < 0.05). Mandal et al. conducted a prospective study and compared the duration of postoperative analgesic efficacy of dexmedetomidine added to pre-emptive infiltration in maxillofacial surgeries [15]. Group DL received 15 mL of 2% lignocaine+adrenaline (1:2,00,000) + 1 µg/kg dexmedetomidine and group PL received 15 mL of 2% lignocaine + adrenaline (1:2,00,000) with normal saline. They concluded that analgesic requirement was significantly earlier in group PL compared to group DL. The results were in concordance with our study. Another randomized trial performed by Mitra et al. observed the analgesic efficacy after wound infiltration with tramadol and dexmedetomidine as an adjuvant to ropivacaine in lumbar discectomies [16]. The median time to first rescue analgesia in group ropivacaine with dexmedetomidine was significantly longer than in group ropivacaine with tramadol and group ropivacaine. Similar observations were also reported in our study. Another randomized double-blind study conducted by Daiki et al. observed the analgesic efficacy of wound infiltration with dexmedetomidine and ropivacaine (RD) group versus ropivacaine (R) group in lumbar spine discectomies [13]. They observed that median time to duration of analgesia was significantly longer in group RD than group R. The results were in agreement with our study.

In the present study, total dose of analgesic requirement was lower in group A (142.50 ± 22.88 mg) compared to group B (197.50 ± 36.76 mg) and was observed to be statistically significant (p < 0.05). Another randomized study was conducted by Mohta et al. to evaluate the analgesic efficacy of local wound infiltration in tubercular spine surgery [17]. Wound infiltration was done with either normal saline (C) group or with 0.375% ropivacaine 3 mg/kg, adrenaline 5 µg/mL, and dexmedetomidine 1 µg/kg on total volume of 0.8 mL/kg (local infiltration analgesia {LIA}) group. They reported that total analgesic requirement was lower in group LIA compared to control group C. The results were in concordance with our study. Kim and Kang conducted a prospective randomized study to compare the effects of perianal infiltration of ropivacaine and dexmedetomidine with ropivacaine for the relief of pain after hemorrhoidectomy [18]. Patients in group C received normal saline and group RO received ropivacaine and group RD received ropivacaine with dexmedetomidine. The analgesia requirement was significantly lower in group RD. Similar observations were also reported in our study. Another randomized study conducted by Singh and Prasad observed the effect of adding dexmedetomidine with bupivacaine for wound infiltration in abdominal hysterectomy and noted that dexmedetomidine in a dose of 1.0 µg/kg provided decreased analgesic demand in postoperative period when compared with wound infiltration via bupivacaine alone [14]. A prospective randomized study was conducted by Kang to evaluate the effects of dexmedetomidine supplemented with pre-emptive ropivacaine infiltration on postoperative pain following inguinal herniorrhaphy [12]. Group RO received 10 mL of 0.2% ropivacaine and group RD received 10 mL of 0.2% ropivacaine with 1 µg/kg dexmedetomidine. They observed that total amount of analgesic consumption was significantly lower in group RD as compared with group RO, similar to our study.

In our study, mean blood pressure was lower in group A than group B during postoperative period and mean difference was statistically significant (p < 0.05). Heart rate was also lower in group A compared to group B at all times postoperatively in the present study. There was statistically significant between the groups (p < 0.05). A prospective study performed by Bhardwaj et al. compared local wound infiltration with ropivacaine alone with ropivacaine plus dexmedetomidine for postoperative pain relief after lower segment cesarean section [19]. Hemodynamic parameters such as heart rate and mean arterial pressure were significantly lower in ropivacaine plus dexmedetomidine group when compared to ropivacaine group at most time intervals (p < 0.05). In our study, patients’ satisfaction score in group A using Likert verbal rating scale was 6 and 7 in eight (26.67%) and 22 (77.33%) patients, respectively. Patients’ satisfaction score in group B using Likert verbal rating scale was 6 and 7 in 23 (76.67%) and seven (23.33%) patients, respectively. Patients' satisfaction score in group A (6.73 ± 45) was higher compared to group B (6.23 ± 0.43) and the difference was highly significant (p < 0.001). A randomized double-blind study performed by Parikh et al. compared dexmedetomidine versus combination of midazolam-fentanyl for tympanoplasty surgery under local anesthesia [20]. They found that the patient’s satisfaction score was better in dexmedetomidine group than midazolam-fentanyl group. The results were in concordance with our study.

The present study had certain limitations. First, we selected the dose of dexmedetomidine taking into consideration the previous studies. However future research with reduced doses in combination with local anesthetics can be done. Second, we did not include any control group receiving placebo. Therefore, a direct comparison between the two groups in this study with a control group could not be instigated. Third, as for lumbar spine surgeries, no clinical studies are available comparing the efficacy of magnesium sulfate when combined with local anesthetic agents, such as bupivacaine and ropivacaine for surgical site infiltration. Further studies are required to be conducted in order to detail the same for abdominal and thoracic surgeries. Fourth, we did not report any related complications in our study, so more studies with larger sample sizes could be done. Fifth, cost-effectiveness of each method had not been studied in our research is another limitation.

## Conclusions

Management of postoperative pain has always been a major task for all surgeons and anesthesiologists, because of the increasing role of outpatient surgery and to facilitate earlier discharge from the hospital. From this study, the authors endorse using ropivacaine plus dexmedetomidine may provide effective analgesia, decreased additional analgesic requirement, and better satisfaction among the patients when compared with ropivacaine-magnesium sulfate wound infiltration during postoperative period after lumbar laminectomy surgeries. This technique can be used as one among the multimodal methods of pain management. Addition of adjuvants such as dexmedetomidine and magnesium sulphate to local anesthetics amplifies the effects of postoperative analgesia in wound infiltration technique. Further research with other local anesthetics with different concentrations can be done in future to expand the role of this technique in postoperative pain management.
